# Serum Levels of Epithelial-Derived Cytokines as Interleukin-25 and Thymic Stromal Lymphopoietin after a Single Dose of Mepolizumab in Patients with Severe Non-Allergic Eosinophilic Asthma: A Short Report

**DOI:** 10.1155/2019/8607657

**Published:** 2019-12-01

**Authors:** Virginija Kalinauskaite-Zukauske, Andrius Januskevicius, Ieva Janulaityte, Skaidrius Miliauskas, Kestutis Malakauskas

**Affiliations:** ^1^Department of Pulmonology, Lithuanian University of Health Sciences, Kaunas, LT-50161, Lithuania; ^2^Laboratory of Pulmonology, Department of Pulmonology, Lithuanian University of Health Sciences, Kaunas, LT-50161, Lithuania

## Abstract

The bronchial epithelium has continuous contact with environmental agents initiating and maintaining airway type 2 inflammation in asthma. However, there is a lack of data on whether reduced airway eosinophilic inflammation can affect the production of epithelial-derived mediators, such as interleukin-25 (IL-25) and thymic stromal lymphopoietin (TSLP). The aim of this study was to investigate the changes in serum levels of IL-25 and TSLP after a single dose of mepolizumab, a humanized monoclonal antibody to interleukin-5 (IL-5), in patients with severe non-allergic eosinophilic asthma (SNEA). We examined 9 SNEA patients before and four weeks after administration of 100 mg mepolizumab subcutaneously. The fractional exhaled nitric oxide (FeNO) level was analysed using an electrochemical assay (NIOX VERO®, Circassia, UK). Serum IL-25 and TSLP levels were measured by ELISA. Four weeks after the single dose of mepolizumab, blood eosinophil count significantly decreased from 0.55 ± 0.20 × 10^9^/l to 0.14 ± 0.04 × 10^9^/l (*p* = 0.01) and FEV_1_ increased from 2.1 ± 0.5 l (65.4 ± 8.8% of predicted) to 2.6 ± 0.4 l (76.4 ± 9.1% of predicted) (*p* = 0.04), while FeNO level has not changed (32.3 ± 8.4 *vs* 42.9 ± 12.6 ppb). Serum IL-25 level significantly decreased from 48.0 ± 17.2 pg/mL to 34.8 ± 17.1 pg/mL (*p* = 0.02) with same tendency in TSLP level: from 359.8 ± 71.3 pg/mL to 275.6 ± 47.8 pg/mL (*p* = 0.02). It has also been noticed a significant relation between changes in the blood eosinophil count and serum IL-25 level (*r* = 0.81, *p* = 0.008), as well as between changes in serum IL-25 and TSLP levels (*r* = 0.93, *p* = 0.004) after a single dose of mepolizumab. Thus, anti-IL-5 treatment with mepolizumab might diminish the production of bronchial epithelial-derived cytokines IL-25 and TSLP in patients with SNEA which is potentially related to reduced eosinophilic inflammation. This trial is registered in ClinicalTrial.gov with identifier NCT03388359.

## 1. Introduction

Asthma is a common, life-lasting airway disease, associated with a high social and economic burden. About 3–8% of all asthma patients have severe asthma, suffering from frequent symptoms and recurrent exacerbations despite the combined treatment with high-dose of inhaled steroids and long-acting bronchodilators, often supplemented with oral steroids [1, 2]. All this leads to a significant loss of life quality and labour productivity, increased mortality risk [3, 4]. The cost of severe asthma treatment represents a significant part of the total cost of all asthma cases [3, 4]. Therefore, severe asthma is the most research-intensive areas of respiratory medicine in the last decade.

Eosinophilic airway inflammation has a key position in the pathogenesis of severe eosinophilic asthma [5, 6]. After activation, eosinophils synthesize a row of cytokines, chemokines, growth factors, and other eosinophil-derived proinflammatory products, and all of them contribute to the airway inflammation in asthma, including airway epithelial cell damage, airway dysfunction, and remodeling [7–9]. Interleukin- 5 (IL-5) is one of the main promoters of eosinophil production, maturation, and release from bone marrow. It also activates eosinophils and prolongs their survival in the circulation, as well as providing an essential signal for their migration into tissue [10]. However, the initial immune response to inhaled air pollutants or other external triggers occurs already in the bronchial epithelium [11–16]. Therefore, dysfunction of epithelial cells is becoming an increasingly important part of the pathogenesis of asthma. There are data that cytokines interleukin- 25 (IL-25) and thymic stromal lymphopoietin (TSLP) are some of the major airway type 2 inflammation regulators derived from the bronchial epithelium [14, 17]. These cytokines have been described as epithelial-derived alarmins that activate and potentiate the inner immune cascade, including airway eosinophilic inflammation, in the presence of actual damage [14, 16–18].

It is unknown whether anti-IL-5-directed treatment affects only eosinophilic inflammation or also other mediators which are involved in airway type 2 inflammation. In this study, we aimed at assessing the changes in serum levels of epithelial-derived mediators as IL-25 and TSLP on mepolizumab, a humanized monoclonal antibody to IL-5, treatment in patients with severe non-allergic eosinophilic asthma (SNEA). We designed to use a single dose of mepolizumab to avoid asthma exacerbations that could influence the intensity of type 2 inflammation, whereas positive drug effect on reduction in blood eosinophils and lung function improvement is observed already after the first dose [19, 20].

## 2. Materials and Methods

### 2.1. Subjects

The study was conducted with the permission of the Regional Biomedical Research Ethics Committee of the Lithuanian University of Health Sciences (BE-2-13) and after signing the informed consent forms. The study was registered in the U.S. National Institutes of Health trial registry ClinicalTrials.gov with identifier NCT03388359.

The study included patients with adult-onset SNEA (the inclusion criteria listed below). Non-allergic asthma was chosen to eliminate allergens as an uncontrollable factor which damage the epithelium and may significantly alter cytokine levels and affect airway type 2 inflammation activity.

The participants were men and women between the ages of 18 and 65 years, recruited from the Department of Pulmonology at Hospital of the Lithuanian University of Health Sciences Kaunas Clinics.

Inclusion criteria were as follows: asthma diagnosis for at least 12 months; non-allergic phenotype, confirmed by the absence of allergy-specific symptoms (watery runny nose or nasal obstruction, conjunctivitis, rashes, urticaria, without dietary restrictions, and any symptoms of digestion) and with negative skin prick tests; blood eosinophil count ≥0.15 × 10^9^/l during the screening visit or documented ≥0.30 × 10^9^/l blood eosinophil count in the 12-month period before the screening denying other possible common causes of eosinophilia (e.g., helminths, allergies); no other reasons that could lead to poor control of asthma symptoms; documented at least 12-month treatment of high doses of inhaled steroids combined with long-acting beta-agonist ± long-acting antimuscarinic agent ± episodic use of oral steroids prior to inclusion in the study; and in the 12 months before the screening visit ≥2 exacerbations of asthma that required treatment with systemic steroids.

The study was open for non-smokers only.

Exclusion criteria included asthma exacerbation, active airway infection, and use of oral steroids 1 month prior to the study; clinically significant permanent allergy symptoms; and treatment with targeted (biological) therapy (e.g., omalizumab, mepolizumab, and benralizumab) at the screening visit.

### 2.2. Study Design

**Figure 1:**
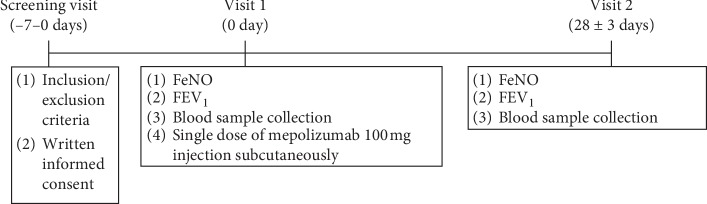
The study design scheme. FEV_1_—forced expiratory volume in 1st second; FeNO—fractional exhaled nitric oxide.

At the screening visit, the inclusion/exclusion criteria were assessed, and informed consent was obtained. During the first study visit, FeNO level was analysed, FEV_1_ was measured, and blood samples were collected for evaluation of blood eosinophil count and epithelial-derived mediators concentrations. After all the procedures, mepolizumab 100 mg was injected subcutaneously. The second study visit was scheduled for 4 weeks. Then, FeNO and FEV_1_ were re-evaluated, and blood samples were re-taken. Only patients without asthma exacerbation during this 4 weeks period were re-evaluated. The study design scheme is presented in Figure 1.

### 2.3. Pulmonary Function Testing

The lung function was evaluated for all study subjects by measuring baseline FEV_1_ using an ultrasonic spirometer (Ganshorn Medizin Electronic, Germany) and compared with the predicted value matched for age, body height, and sex according to the standard methodology. FEV_1_ was measured three times and recorded only the highest of three reproducible measurements.

### 2.4. FeNO Measurement

All study subjects underwent FeNO analysis with an online method using a single-breath exhalation and an electrochemical assay (NIOX VERO®, Circassia, UK), according to ATS-ERS guidelines [21]. Patients made an inspiration of eNO-free air via a mouthpiece immediately followed by full exhalation at a constant rate (50 mL/sec) for at least 10 seconds. The mean of three readings at the end of the expiration (plateau phase) was taken as the representative value for each measurement.

### 2.5. Skin Prick Test

All study subjects were screened for allergies by the skin prick test at least 6 months prior to enrollment. Standardized allergen extracts (Stallergenes S.A., France) were used for the following allergens: *Dermatophagoides pteronyssinus*, *Dermatophagoides farinae*, cat and dog dander, mixture of pollen of 5 grasses, birch pollen, mugwort, *Alternaria*, *Aspergillus*, and *Cladosporium*. Negative control was performed with diluent (saline) and positive control with histamine hydrochloride (10 mg/mL). Results of the test were evaluated 15 min after application. The skin prick test was estimated to be positive when the mean wheal diameter reaches ≥3 mm.

### 2.6. Detection of Protein Level by Investigating Individuals' Blood Serum Samples

Protein (IL-25 and TSLP) levels in blood serum samples were measured by the enzyme-linked immunosorbent assay (ELISA) according to the manufacturer's instructions. The following ELISA kits were used for experiments: IL-25 (R&D Systems, USA) lower limit of detection (LLD)—11.7 pg/mL and TSLP (R&D Systems, USA) LLD—7.8 pg/mL. 100 *μ*l of serum samples was used for experiments. Blood was collected into BD Vacutainer® SST™ II Advance Blood Collection Tubes and allowed to clot for 30 min. After that, the tubes were centrifuged at 1300 ×g 10 min at room temperature to separate serum from clotted blood. Serum immediately was collected and divided into 1 mL cryogenic tubes that were frozen in −80°C for further proteins level analysis. ELISA measurements were performed after a sufficient amount of samples was collected. The results were expressed as protein concentration per 1 mL of serum.

### 2.7. Statistical Analysis

Statistical analysis was performed by using GraphPad Prism 6 for Windows (ver. 6.05, 2014; GraphPad Software Inc., San Diego, CA). Protein concentration data were represented as the mean ± standard error of the mean or with specific values for each subject, including the overall mean. Significant differences between two dependent groups were determined using the Wilcoxon matched-pairs signed-rank test. Spearman rank correlation coefficient was used to evaluate correlations. *p* < 0.05 was considered as statistically significant.

## 3. Results and Discussion

### 3.1. Characteristics of Study Population

In this study, we examined 9 patients with adult-onset SNEA at two time points: before and 4 weeks after 100 mg subcutaneous injection of mepolizumab. The subjects with SNEA were middle or elderly aged with a tendency to overweight, on the Global Initiative for Asthma (GINA) step 4-5 treatment, and had impaired lung function, increased blood eosinophil count, and elevated FeNO level (more detailed data are presented in Table 1). Any patient experienced asthma exacerbation up to a second visit. Four weeks after a single dose of mepolizumab, significant reduction of the blood eosinophilia and improvement in lung function were observed, while FeNO level remained stable (Table 1).

### 3.2. Changes in Blood Eosinophil Count and Serum IL-25 and TSLP Levels after a Single Dose of Mepolizumab

**Figure 2:**
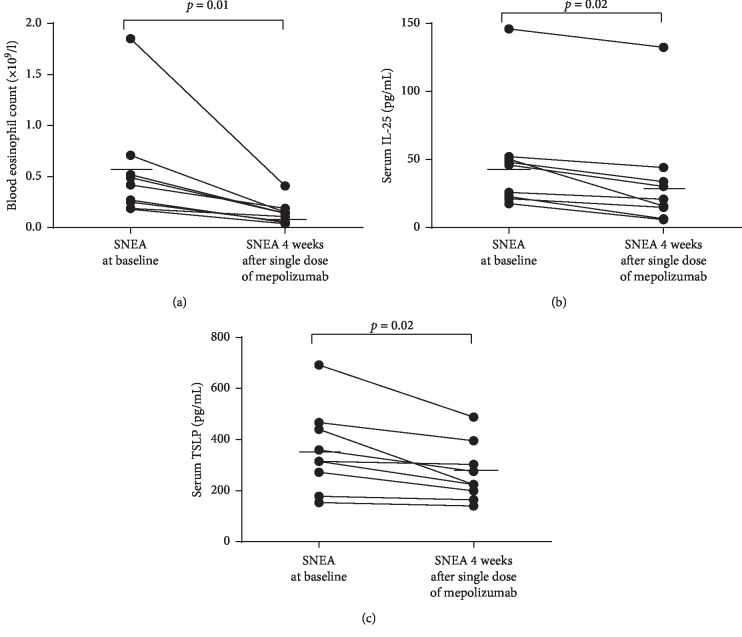
Changes in blood eosinophil count and serum interleukin-25 (IL-25) and thymic stromal lymphopoietin (TSLP) level in patients with severe non-allergic eosinophilic asthma (SNEA) 4 weeks after a single dose of mepolizumab (*n* = 9). (a) Blood eosinophil count. (b) IL-25. (c) TSLP. Statistical analysis—Wilcoxon matched-pairs signed-rank test. Data are presented with specific values for each subject, including the overall mean.

Four weeks after the single dose of mepolizumab, blood eosinophil count significantly decreased from 0.55 ± 0.20 × 10^9^/l to 0.14 ± 0.04 × 10^9^/l (*p*=0.01, Figure 2(a)). In this study, we found that the serum level of IL-25 was significantly reduced from 48.0 ± 17.2 pg/mL to 34.8 ± 17.1 pg/mL already after a single dose of add-on treatment with mepolizumab (*p*=0.02, Figure 2(b)). The change in serum TSLP level was similar: 359.8 ± 71.3 pg/mL, before mepolizumab administration, and 275.6 ± 47.8 pg/mL (*p*=0.02), 4 weeks after a single dose of mepolizumab (Figure 2(c)).

**Figure 3:**
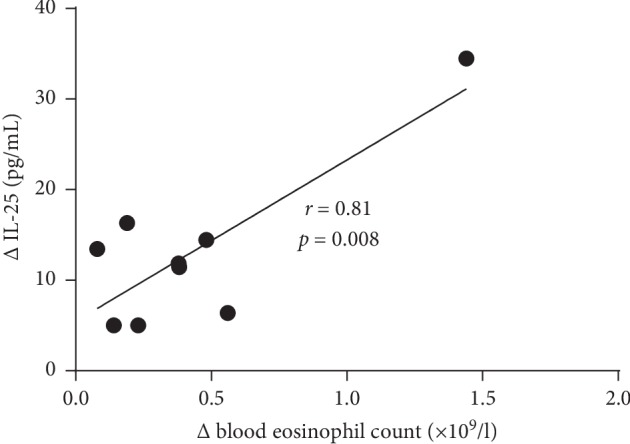
Correlation between changes in the blood eosinophil count and serum interleukin-25 (IL-25) level 4 weeks after a single dose of mepolizumab in patients with severe non-allergic eosinophilic asthma (*n* = 9). *r*—Spearman rank correlation coefficient.

**Figure 4:**
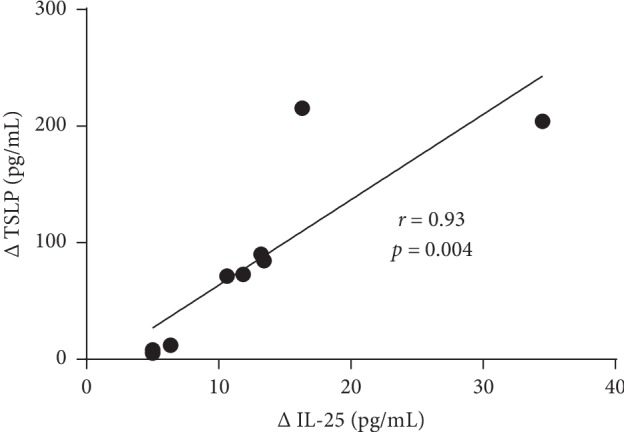
Correlation between changes in serum interleukin-25 (IL-25) and thymic stromal lymphopoietin (TSLP) level in patients 4 weeks after a single dose of mepolizumab with severe non-allergic eosinophilic asthma (*n* = 9). *r*—Spearman rank correlation coefficient.

Significant correlations were observed between changes in the blood eosinophil count and serum IL-25 level (*r* = 0.81, *p*=0.008) (Figure 3), as well as between changes in serum IL-25 and TSLP levels (*r* = 0.93, *p*=0.004) (Figure 4). However, the correlation between changes in the blood eosinophil count and serum TSLP level was not significant (*r* = 0.49, *p* > 0.05).

## 4. Discussion

In this study, attention was drawn to the bronchial epithelium dysfunction and how the level of epithelial-derived cytokines changes during treatment with anti-IL-5 drug when eosinophilic inflammatory activity is reduced. It was found that a single dose of mepolizumab in patients with SNEA significantly decreased blood eosinophil count and improved lung function, whereas FeNO did not. We have also noticed that anti-IL-5 treatment reduced the serum level of epithelial-derived cytokines, such as IL-25 and TSLP.

Blood eosinophil depletion and lung function improvement (measured by FEV_1_) during treatment with mepolizumab have been demonstrated in many studies to confirm the appropriateness of mepolizumab during severe eosinophilic asthma [19, 22–24]. Our study does not contradict this, suggesting that patients have been appropriately selected and that the given treatment effectively inhibits eosinophilic inflammation, assuming that the newly identified results—changes in IL-25 and TSLP—are associated with the action of mepolizumab.

IL-25 and TSLP are epithelial-derived cytokines that play an essential role in stimulating Th2 cytokine response and initiating airway type 2 inflammation in asthma [16–18]. By investigating eosinophilic asthma pathogenesis, it was noted that IL-25 significantly influences the production of type 2 cytokines, including IL-5, essential for eosinophilic inflammation [25–27]. Analogous results were obtained with the TSLP [28–31]. There is evidence that these cytokines are related to asthma severity. It is found that the amount of IL-25 in the sputum was correlated with the severity of asthma—the highest level of IL-25 in sputum was found in severe asthma [28]. The significant changes in TSLP protein expression were identified in lung tissue, and an inverse correlation with the severity of bronchial obstruction was observed [29]. The importance of IL-25 and TSLP in eosinophilic asthma is increasingly being explored. The change in their production is believed to play an essential role in the pathogenesis of asthma by initiating airway type 2 inflammation and promoting the production of type 2 cytokines [6, 17, 18, 30, 32]. However, no studies have yet been carried out to evaluate the effect of anti-IL-5 treatment on the bronchial epithelium-derived cytokines. We found that reduced activity of eosinophilic inflammation with mepolizumab affects the level of epithelial-derived cytokines IL-25 and TSLP and it was associated with changes in blood eosinophil count. How the reduction of eosinophilic inflammation is associated with epithelial cytokine level has only speculations. The bronchial epithelium has the naïve bronchial epithelial cells, which produce some levels of IL-5 [31]. Animal studies have shown that bronchial epithelial cells, isolated from mice with OVA-induced allergic airway disease, produced elevated levels of IL-5 mRNA and protein as compared to bronchial epithelial cells from naïve mice. Therefore, IL-5 produced by epithelial cells contributed to mucous metaplasia and airway eosinophilia and can impact the microenvironment of the lung, modifying pathologic and protective immune responses in the airways [31]. Thus, bronchial-epithelial eosinophilia leads to the production of an additional amount of IL-5, and it is an important element for supporting eosinophilic inflammation. In addition, eosinophils release granular proteins that can damage the bronchial epithelial cells [33] and thus lead to higher epithelial-derived cytokine production. In terms of direct effects of IL-5 on bronchial epithelial cells, there are data that differentiated human airway epithelial cells express functional IL-5 receptors and that this cytokine may promote epithelial cell growth and proliferation (at the same time also affect the production of cytokines) [34]. In response to exogenous stimuli, epithelial-derived IL-25 and TSLP together with other bioactive substances elicit innate lymphoid cell (ILC2) responses in the lungs [6, 17]. Activated ILC2s can subsequently promote IL-5-mediated eosinophil recruitment and produce large amounts of Th2 cytokines, including IL-5, which enhances eosinophil adhesion to bronchial epithelial cells. ILC2s also produce amphiregulin, which promotes the repair of the airway epithelium [10]. Collectively, all these findings suggest that IL-5 affects airway physiology in asthma in part through effects on airway epithelial cells [34]. Thus, the hypothesis is that by blocking IL-5, mepolizumab selectively inhibits both the inner cascade IL-5 and the epithelial-derived IL-5, aggravate eosinophil adhesion to bronchial epithelial cells, reduce its infiltration with eosinophils and their degranulation, and thus reduce the eosinophilic inflammation and subsequent epithelial damage, as well as epithelial cytokine production. This is reinforced by us obtaining a significant correlation between changes in the blood eosinophil count and serum IL-25 level and changes in serum IL-25 and TSLP levels after a single dose of anti-IL-5 treatment with mepolizumab in patients with SNEA.

According to the study data, blood levels of certain cytokines are unlikely to accurately reflect the processes in the lungs as we did not find a reliable correlation between changes in TSLP and blood eosinophil count. These insights are also observed in a study with atopic dermatitis, where serum TSLP level did not significantly correlate with disease severity, blood eosinophil counts, and serum total immunoglobulin E levels, suggesting that TSLP does not mainly enter the blood circulation [35] or related to how acute the disease is. There are data that the early exaggerated production of TSLP might be important for initiating immune processes but may not be through serum TSLP [36]. However, the importance of TSLP as a key cytokine for epithelial damage has been demonstrated by a significant correlation with IL-25.

Additionally, FeNO is attributed to eosinophilic inflammatory biomarkers, and the relationship with eosinophilia and FeNO level is established [23], but, according to studies, no significant changes were found in FeNO level on eosinophilic inflammation-reducing treatment [37, 38]. The results of our study were analogous. However, FeNO is referred to as a validated eosinophilic biomarker and its stability, despite suppressed eosinophilic inflammation, is more difficult to understand. FeNO can be influenced by many factors, including inhaled corticosteroids and body mass index [39–42]. It is also associated with interleukin- 4 and interleukin- 13, but these cytokines are crucially important in allergic asthma cases [42, 43]. In our study, SNEA patients received inhaled steroids in high doses and had a slightly increased body mass index; however, they were all non-atopic. Therefore, in the case of severe asthma, the assessment of FeNO as a marker of eosinophilic inflammatory activity may not be valuable but is likely more sensitive to withdrawal of steroids [44].

The study is limited by the lack of a control group for comparison of the effect of mepolizumab on the levels of epithelial-derived cytokines. However, this was a brief observation study for 4 weeks to minimize the risk of exacerbations and course variability of asthma that could have an impact on TSLP and IL-25 production. Thus, only clinically stable severe asthma patients, free of systemic steroids at least 1 month before the study, were included and patients without asthma exacerbation during 4 weeks period after a single dose of mepolizumab were re-evaluated. Additionally, all study subjects were non-atopic and non-smokers, so it could be presumed that contact with inhaled non-specific air particles, which trigger the epithelium, is constant.

## 5. Conclusion

Our study results demonstrate that anti-IL-5 treatment with mepolizumab might diminish the production of bronchial epithelial-derived cytokines IL-25 and TSLP in patients with SNEA which is potentially related to reduced eosinophilic inflammation. These findings indicate that mepolizumab by modulation of bronchial epithelium function could provide a broader impact on the severe eosinophilic asthma pathophysiology. This may be important when making clinical decisions for patients non-responsive to other biologics for severe refractory asthma.

## Figures and Tables

**Table 1:** Demographic and clinical characteristics of the study population.

	SNEA subjects	
Number (*n*)	9	
Sex (M/F)	5/4	
Age (years)	53 ± 5.2	
BMI (kg/m^2^)	28.9 ± 1.6	
High-dose iCS + LABA	5	
High-dose iCS + LABA + LAMA	3	
High-dose iCS + LABA + theophylline	1	

	At baseline visit	4 weeks after a single dose of mepolizumab
FEV_1_ (l)	2.1 ± 0.5	2.6 ± 0.4^*∗*^
FEV_1_ (% of predicted)	65.4 ± 8.8	76.4 ± 9.1^*∗*^
Blood eosinophil count (×10^9^/l)	0.55 ± 0.20	0.14 ± 0.04^*∗*^
FeNO (ppb)	32.3 ± 8.4	42.9 ± 12.6

F: female; M: male; FEV_1_: forced expiratory volume in 1st second; FeNO: fractional exhaled nitric oxide; iCS: inhaled glucocorticosteroids; LABA: long-acting *β*-adrenoceptor agonists; LAMA: long‐acting muscarinic antagonists. Data are presented as the mean ± standard error of the mean. ^*∗*^*p* < 0.05 comparing with the baseline visit.

## Data Availability

The study data used to support the findings of this study are available from the corresponding author upon request.

## Abbreviations

ELISA: Enzyme-linked immunosorbent assay
FeNO: Exhaled nitric oxide concentration
FEV_1_: Forced expiratory volume in 1st second
GINA: Global Initiative for Asthma
ILC2: Type 2 innate lymphoid cells
IL-5: Interleukin-5
IL-25: Interleukin-25
LLD: Lower limit of detection
SNEA: Severe non-allergic eosinophilic asthma
TSLP: Thymic stromal lymphopoietin.
